# Superior diagnostic performance of perfusion-cardiovascular magnetic resonance versus SPECT to detect coronary artery disease: The secondary endpoints of the multicenter multivendor MR-IMPACT II (Magnetic Resonance Imaging for Myocardial Perfusion Assessment in Coronary Artery Disease Trial)

**DOI:** 10.1186/1532-429X-14-61

**Published:** 2012-09-02

**Authors:** Juerg Schwitter, Christian M Wacker, Norbert Wilke, Nidal Al-Saadi, Ekkehart Sauer, Kalman Huettle, Stefan O Schönberg, Kurt Debl, Oliver Strohm, Hakan Ahlstrom, Thorsten Dill, Nadja Hoebel, Tamas Simor

**Affiliations:** 1 Cardiology, University Hospital Lausanne, Rue de Bugnon 46, CH-1011, Lausanne, Switzerland; 2 University Hospital Wuerzburg, Wuerzburg, Germany; 3 University of Florida Health Science Center, Gainesville/Jacksonville, USA; 4 Franz-Volhard Clinic-Humboldt University, Berlin, Germany; 5 Landshut Hospital, Landshut, Germany; 6 Semmelweis University Hospital, Budapest, Hungary; 7 LMU Munich, Grosshadern, Germany; 8 current affiliation - University Medical Center Mannheim, Mannheim, Germany; 9 University Hospital Regensburg, Regensburg, Germany; 10 St. Gertrauden Hospital Berlin, Berlin, Germany; 11 Uppsala University Hospital, Uppsala, Sweden; 12 Kerckhoff Clinics Bad Nauheim, Nauheim, Germany; 13 Current affiliation - Sana Kliniken Duesseldorf, Duesseldorf, Germany; 14 GE Healthcare Buchler GmbH & Co.KG, Munich, Germany; 15 Medical University of Science, Pecs, Hungary

**Keywords:** Cardiovascular magnetic resonance, Scintigraphy, Coronary disease, Perfusion, Ischemia

## Abstract

**Background:**

Perfusion-cardiovascular magnetic resonance (CMR) is generally accepted as an alternative to SPECT to assess myocardial ischemia non-invasively. However its performance vs gated-SPECT and in sub-populations is not fully established. The goal was to compare in a multicenter setting the diagnostic performance of perfusion-CMR and gated-SPECT for the detection of CAD in various populations using conventional x-ray coronary angiography (CXA) as the standard of reference.

**Methods:**

In 33 centers (in US and Europe) 533 patients, eligible for CXA or SPECT, were enrolled in this multivendor trial. SPECT and CXA were performed within 4 weeks before or after CMR in all patients. Prevalence of CAD in the sample was 49% and 515 patients received MR contrast medium. Drop-out rates for CMR and SPECT were 5.6% and 3.7%, respectively (ns). The study was powered for the primary endpoint of non-inferiority of CMR vs SPECT for both, sensitivity and specificity for the detection of CAD (using a single-threshold reading), the results for the primary endpoint were reported elsewhere. In this article secondary endpoints are presented, i.e. the diagnostic performance of CMR versus SPECT in subpopulations such as multi-vessel disease (MVD), in men, in women, and in patients without prior myocardial infarction (MI). For diagnostic performance assessment the area under the receiver-operator-characteristics-curve (AUC) was calculated. Readers were blinded versus clinical data, CXA, and imaging results.

**Results:**

The diagnostic performance (= area under ROC = AUC) of CMR was superior to SPECT (p = 0.0004, n = 425) and to gated-SPECT (p = 0.018, n = 253). CMR performed better than SPECT in MVD (p = 0.003 vs all SPECT, p = 0.04 vs gated-SPECT), in men (p = 0.004, n = 313) and in women (p = 0.03, n = 112) as well as in the non-infarct patients (p = 0.005, n = 186 in 1–3 vessel disease and p = 0.015, n = 140 in MVD).

**Conclusion:**

In this large multicenter, multivendor study the diagnostic performance of perfusion-CMR to detect CAD was superior to perfusion SPECT in the entire population and in sub-groups. Perfusion-CMR can be recommended as an alternative for SPECT imaging.

**Trial registration:**

ClinicalTrials.gov, Identifier: NCT00977093

## Background

Early detection of coronary artery disease (CAD) and in particular of myocardial ischemia remains a major challenge even with the advent of novel non-invasive imaging techniques and further development of existing modalities. An increasing number of cardiovascular magnetic resonance (CMR) studies documented a high diagnostic performance of perfusion-CMR vs conventional x-ray coronary angiography (CXA) [1-9] and showed its prognostic value [10,11]. In comparison with CXA, for both, perfusion-CMR [12,13] as well as for SPECT, cost-effectiveness was demonstrated [14,15]. However, for several sub-groups of patients the diagnostic performance of perfusion-CMR and its potential superiority over SPECT is not well established. The first study of the MR-IMPACT program [2] designed for dose-finding was the largest perfusion-CMR trial at its time and demonstrated equal performance vs SPECT in the head-to-head comparison, and demonstrated superiority of CMR when compared versus the entire SPECT population. The MR-IMPACT II was designed to compare the diagnostic performance of CMR vs SPECT for the detection of CAD (defined as ≥50% diameter reduction of coronary vessels in CXA) in a large international multicenter, multivendor design at a fixed contrast medium (CM) dose. The primary end-point of MR-IMPACT II was the comparison of sensitivity and specificity of perfusion-CMR to detect CAD on CXA vs SPECT based on a single-point threshold reading. In this comparison, perfusion-CMR was more sensitive, but less specific for the detection of CAD in comparison with SPECT [16]. This single-threshold reading assesses diagnostic performance on a single point on the ROC curve, thus, rendering results susceptible for the reading threshold [17]. The comparison of test performances by means of the areas under the ROC curves (AUC) avoids such potential bias [17]. Therefore, we analyzed as a pre-defined secondary end-point of the MR-IMPACT II the AUCs for perfusion-CMR and SPECT for the entire study population. Additional sub-group analyses assessed the diagnostic performance in patients studied by gated-SPECT, in patients without prior myocardial infarctions (MI) with single- or multi-vessel disease, as well as in men and in women. In addition, the primary end-point was also recalculated for a single-point reading at the optimum threshold as derived from the AUC analyses.

## Methods

### Study design and patient population

This phase III clinical trial was conducted at 33 centers in Europe and the US. Eligible patients were those scheduled for a conventional CXA and/or a SPECT examination for clinical reasons. Before study entry all patients had to agree to undergo all 3 imaging studies. As no interventions were allowed on the coronary arteries in the time period between the 3 tests, most patients underwent CXA as the last test, i.e. after having had the CMR and SPECT study. Exclusion criteria were: Acute MI (<2 weeks prior to study enrolment), history of coronary artery bypass grafting, unstable angina pectoris, decompensated heart failure, contraindications for adenosine and/or CM, and severe arrhythmias (atrial fibrillation, bigeminus, >15 extrasystoles/min) considered to compromise quality of CMR imaging. The study was conducted according to the Declaration of Helsinki, the principles of Good Clinical Practice, and was approved by the Health Authorities and the local Ethics Committee of each participating institution. All patients gave written informed consent before study participation.

### Efficacy measures

As a pre-defined secondary endpoint of the trial, the AUCs for perfusion-CMR and SPECT were compared for the assessment of the diagnostic performances of CMR and SPECT to detect CAD thereby determining test performances over a range of thresholds [17]. In addition, the ROC approach was also used to assess the test performances in multi-vessel disease (MVD) patients, in men and in women, as well as in the patient population without prior MI. As reported elsewhere, for the primary end-point of MR-IMPACT II a single threshold reading was used to assess sensitivity and specificity of CMR and SPECT to detect CAD [16].

#### Standard of reference - definition of CAD

Two criteria were combined to define CAD by the standard of reference: 1). The presence of a ≥50% diameter stenosis in quantitative coronary angiography in 2 orthogonal planes (≥75% area reduction) as was used in previous studies [1-3,18] present in ≥1 coronary artery of ≥2 mm diameter using a core laboratory (Cleveland Clinic Foundation, Cleveland, USA). This criterion accounted for 94.6% of all CAD positive patients in this study. 2). The history of a previous MI was considered. Thus, patients with a history of MI were categorized as CAD positive even in the absence of stenosed coronary arteries (e.g. after coronary artery stenting in the setting of acute MI). This criterion allowed to correctly assign patients with a perfusion deficit (=hypoperfusion of scar tissue) to the group of CAD positive patients. This criterion accounted for 5.4% of patients with CAD. Conversely, patients with a history of successful PCI/stenting (with a residual stenosis ≤50% in the actual CXA) and without a history of MI do not fulfill the definition of CAD (and are assumed to yield normal perfusion studies). For the sub-analysis of patients without MI, the patients with a history of prior MI were excluded. Vessels of <2 mm diameter were not considered for definition of CAD, since such small vessels are in general not revascularized (e.g. no stents available for <2 mm vessels).

#### CMR

In 1.5 T scanners of various vendors breath-hold MR first-pass perfusion examinations were performed. The patients had to refrain from coffee, tea, chocolate or other caffeinated beverages and food for at least 24 h before the CMR exam. After 3 minutes of an adenosine infusion (0.14 mg/min/kg IV) a bolus of 0.075 mmol/kg Gd-DTPA-BMA (Omniscan, GE Healthcare, US) was injected into a peripheral vein with power-injectors at 5 ml/sec (followed by a 25 ml saline flush) during a breath-hold. A CM dose of 0.075 mmol/kg was chosen according to recommendations of the food and drug administration (FDA) to test the minimal effective dose. During bolus arrival, 3 short-axis slices were acquired every heart beat at ¼, ½, and ¾ of the left ventricular (LV) long axis (non-slice selective 90°-preparation, fast gradient-echo acquisition with an echo-planar component where available; spatial resolution: 2-3 mm x 2-3 mm, slice thickness 8-10 mm). At the same locations, at 10 and 25 minutes after the stress imaging, a rest perfusion imaging at the same CM dose and a late enhancement study (with the inversion time nulling normal myocardium) were performed, respectively.

### Perfusion-CMR analyses

CMR data were analyzed visually by 3 blinded readers in an independent core laboratory (Independent Review Center, GE Healthcare, former Nycomed Amersham Imaging, Princeton, USA). The 3 readers were blinded with respect to any clinical information of the patients or results of the other examinations.

The ROC analysis was used to compare the diagnostic performance of CMR and SPECT evaluating the test performance over the full range of thresholds. For the ROC analysis, 16 segments/heart (represented by bulls eyes not including apical segment 17) were graded each as showing severely abnormal stress perfusion (=3; defined as myocardium being black at the peak bolus), or moderately abnormal defect (=2; myocardium being dark grey), borderline abnormal defect (=1; myocardium being light grey), or normal perfusion (=0; myocardium being bright). Additional criteria indicative for true hypoperfusion vs. artifacts were subendocardial signal reduction persisting longer than the CM first-pass through the LV cavity, signal reduction in several slices and neighboring regions [2]. For this ROC analysis the gradings of all 16 segments of a heart were summed up and the resulting gradings of the 3 readers were averaged (referred to as “summed grading”) to enter the ROC analyses (e.g. individual gradings of a study by readers 1–3 of 14, 18, 16 yield a “summed grading” of 16 for this study). Of note, the grading categories 0–3 were not used to assess the data quality, but severity of perfusion defects. Regarding data quality (predominantly determined by the presence or absence of breathing motion and triggering artifacts), a patient was excluded from analyses, if all 3 readers assessed ≥1 segment as non-diagnostic in a patient with all other segments normal. If only 1 or 2 readers found non-diagnostic segments, the gradings of the readers which judged all segments as diagnostic were averaged and thus, these examinations were kept in the analyses. These criteria were implemented to avoid a selection bias towards high-quality data. In analogy to the SPECT readings (of stress and rest, i.e. redistribution images), the blinded readers for CMR were presented with the stress perfusion images together with the late enhancement images (i.e. redistribution images) demonstrating viable and scar tissue. As in previous trials [1-3], the summed gradings were calculated from the stress perfusion images only, since differentiation into ischemia and scar tissue was not the primary aim of the study.

To demonstrate the dependence of the primary endpoint analysis on the selection of threshold, the sensitivity and specificity scores were also calculated for the thresholds at summed gradings of 19, 21, and 23, which were located at the upper left portion of the ROC curve.

#### SPECT

Stress and rest SPECT examinations were performed as reported elsewhere [16] according to generally accepted guidelines [19] on machines of different vendors (2 or 3 head cameras) with ^99m^Tc- or ^201^Tl-tracers, adenosine dose as for perfusion-CMR, or physical stress, and 1 or 2 days protocols. The patients had to refrain from coffee, tea, chocolate or other caffeinated beverages and food for at least 24 h before the SPECT exam. Gated-SPECT using ^99m^Tc-tracers was strongly recommended, but ungated acquisitions and/or ^201^Tl-tracers were accepted if part of the performing institution’s clinical routine. In the efficacy population, i.e. all 3 methods completed, gated-SPECT was performed in 253 patients. ^201^Tl-tracer was used in 32 patients (rest and stress) and in 8 additional patients for rest studies only (6.9% and 1.7%, respectively). Algorithms for attenuation correction or resolution recovery were not applied as these were not available or not identical over all sites.

SPECT data were analyzed visually by 3 blinded readers using a core laboratory (Beacon Bioscience, Inc. Doylestown, USA). The 3 readers were blinded with respect to any clinical information of the patients or results of the other examinations. Each reader was presented with 10–12 short-axis as well as 6–9 vertical and horizontal long-axis images for both, stress and rest condition. Gated-SPECT data were also presented to the readers, if they had been acquired. For the ROC analysis perfusion deficits were graded in each of the 16 segments as fully reversible (=3), partially reversible (=2), fixed defect (=1), or normal (=0), and summed gradings (averaged for the 3 readers) were calculated as for the CMR analyses. If all 16 segments were normal (summed grading = 0), but other pathologies were present such as transient ischemic dilation or exclusive apical ischemia a summed grading of 3 was assigned. Patients with ≥1 segment graded as non-diagnostic were treated as for the CMR examination. The primary endpoint results (=binary readings according regulatory requirements) were reported previously [16].

#### Statistical analysis

Sample size calculations for the primary endpoints were reported previously [16]. For the comparison of CMR vs. SPECT by ROC analysis, estimates suggested a required sample size of ~370 patients to yield an 90% power to detect a difference in AUC of 0.10 (AUC 0.75 vs. 0.85 for SPECT and CMR, respectively [2]) at a one-sided P-value of 0.05 [20]. Unlike the sensitivity-specificity approach used for the primary endpoint, the ROC analysis approach used for the secondary endpoints assesses sensitivity and specificity “simultaneously” thereby taking into account, that sensitivity and specificity of a test are inversely related [17].

ROC analyses were performed for both modalities on a patient basis (Rockit 0.9.1Beta). AUCs for CMR and SPECT were compared by a univariate z-score test (null hypothesis: data sets arose from binormal ROC curves with equal areas beneath them). In addition to the analyses of all patients (1–3 vessel disease), MVD patients were analyzed by exclusion of patients with single vessel disease. AUCs were also calculated for the male and female populations separately. After exclusion of patients with prior MI, AUCs were calculated for the entire study population and the MVD population. All tests were two-sided and a p-value <0.05 was considered statistically significant.

## Results

### Patient characteristics

From the 533 patients enrolled 515 received the MR CM and entered the safety analysis (the frequency of mild to moderate adverse events was 22.1% occurring in 74 patients). As reported earlier, this MR-IMPACT II confirms the high safety profile of the CMR technique, as no severe adverse events occurred among the 515 patients [16].

**Figure 1:**
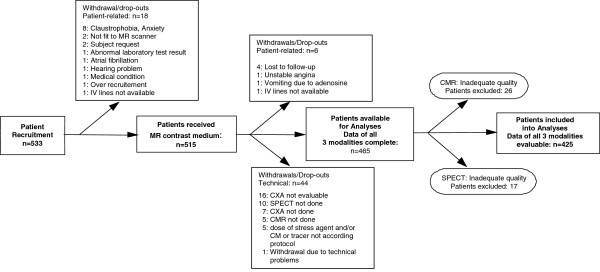
**Flow Chart.** Flow chart demonstrating the number of eligible patients and drop-outs. CMR: cardiovascular magnetic resonance; CM: contrast medium (Gd-DTPA-BMA); CXA: coronary X-ray angiography; Pats: patients. SPECT: single-photon-emission-computed-tomography.

Of the 465 patients with data of all 3 modalities complete (Table 1 and Figure 1), 227 (48.8%) had coronary artery stenoses with ≥50% diameter reduction, 73 had occlusions (15.7%), 129 (27.7%) had infarctions, and 25 patients (5.4%) of those with infarctions showed no significant stenoses (<50% diameter reduction) on CXA. Prevalence of CAD in the population without a history of infarction was 29%. No patients of the previous MR-IMPACT I were included in the analyses of MR-IMPACT II.

**Table 1:** Demographics of study population

	**n (%)**
Patients enrolled and CM administered:^1^	515
Male sex	377 (73.2)
Age (mean±SD)	60±10.3 years
Body mass index (mean±SD)	28.2±4.3 kg/m^2^
Risk factors:	
Hypertension	358 (69.5)
Hypercholesterolemia	354 (68.8)
Diabetes	92 (17.8)
History of heart failure	106 (20.6)
Myocardial infarction	139 (27.0)
Percutaneous coronary intervention (PCI)	170 (33.0)
Angina pectoris	414 (80.4)
CCS I	87 (16.9)
CCS II	227 (44.1)
CCS III	46 (8.9)
CCS IV	21 (4.1)
Patients with all 3 test data sets complete (efficacy population):	n 465
Coronary artery disease	227 (48.8)
Left main	14 (3.0)
LAD	134 (28.8)
LCX	104 (22.4)
RCA	112 (24.1)
Multivessel disease	113 (24.3)
Myocardial infarction	129 (27.7)
Medication:	
Any drugs	496 (96.4)
Beta-blockers	367 (71.3)
Lipid lowering	354 (68.8)
Angiotensin-converting enzyme inhibitors	306 (59.4)
Diuretics	131 (25.5)
Calcium channel blockers	99 (19.2)
Antithrombotic	425 (82.6)
MR – not evaluable	26 (5.6)
SPECT – not evaluable	17 (3.7)

CCS: Canadian Cardiovascular Society; LAD: left anterior descending coronary artery; LCX: left circumflex coronary artery; RCA: right coronary artery.

^1^ Eighteen patients were recruited but did not receive MR contrast medium, for reasons, see Flow chart in Figure 1.

### Results of ROC analyses

**Figure 2:**
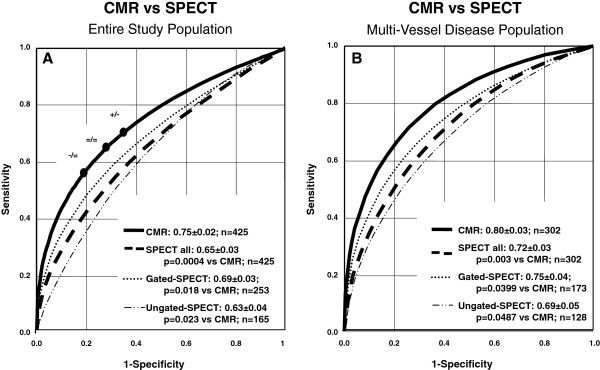
**Diagnostic performance in the entire study population – ROC analyses.** Diagnostic performance of perfusion-CMR and SPECT imaging compared by receiver operating characteristics curves (ROC) analyses for detection of CAD (per patient analysis). A). CMR performs superior to all SPECT studies in 1–3 vessel disease (1–3 VD) patients and is also superior to the gated-SPECT and ungated-SPECT groups. Difference in AUC between gated-SPECT and ungated-SPECT did not reach statistical significance. The dots on the ROC curve for CMR indicate the sensitivities and specificities for various thresholds (i.e. at summed gradings of 23 [dot on the left], 21 [middle dot], and 19 [dot on the right]) with + and – indicating superiority and inferiority vs SPECT, respectively, and = indicating non-inferiority versus SPECT for both, sensitivity and specificity, as defined as primary end-point of the study (for details on the definition of the primary endpoint see reference [16]). These dots located at various reading thresholds illustrate that comparisons for sensitivity and specificity depend on the thresholds applied for the 2 tests. Thus, for the same data set, superiority or inferiority can be obtained (for sensitivity or specificity comparisons) depending on the reading thresholds used. Reading CMR studies at a high threshold for perfusion deficits (point on the left on the ROC curve) yields CMR inferiority for sensitivity and CMR non-inferiority for specificity vs SPECT, while the same CMR test read with a low threshold (point on the right of the ROC curve) yields a superior sensitivity for CMR vs SPECT with inferiority for specificity. This dependence of comparisons upon reading thresholds is eliminated by the ROC approach, which assesses test performance over the entire range of reading thresholds. B): Perfusion-CMR is superior to SPECT in multi-vessel disease patients. Sub-group analyses for gated-SPECT and ungated-SPECT yielded superiority for CMR, as well.

Twenty-six CMR studies (5.6% of 465) and 17 SPECT studies (3.7% of 465, p = 0.21 vs CMR) were deemed non-evaluable by the MR and SPECT readers, respectively (Figure 2). Prevalence of CAD in the entire study population was 60% and 39% for men and women, respectively (p = 0.0002).

**Figure 3:**
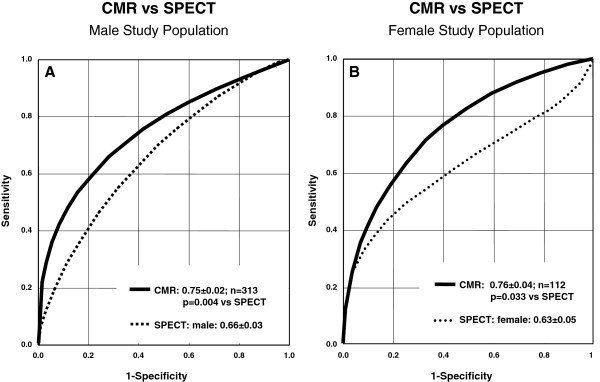
**Diagnostic performance in men and women – ROC analyses.** Perfusion-CMR is superior vs SPECT in both, men (A) and women (B). Numbers indicate mean ± standard error of the AUC.

The ROC analyses as shown in Figure 2A demonstrate a higher AUC, i.e. a higher diagnostic performance, of CMR vs SPECT (entire population: 0.75 ± 0.02 vs 0.65 ± 0.03,p = 0.0004) and vs gated-SPECT (0.69 ± 0.03, p = 0.018, ^201^Tl-studies excluded). Similar results were obtained for the MVD population as shown in Figure 2B. Figure 3A/B shows superiority of perfusion-CMR over SPECT in men and women, respectively. Perfusion-CMR was also superior versus gated-SPECT in the patient population without prior MI (CMR: AUC 0.69 ± 0.04 vs 0.57 ± 0.04, p = 0.0054, n = 186 in all non-MI patients; CMR: AUC 0.78 ± 0.04 vs 0.65 ± 0.05, p = 0.015, n = 140 in MVD non-MI patients, ^201^Tl-studies excluded). Similar AUCs were obtained for the different readers ranging from 0.72 to 0.83 for the CMR readers and from 0.63 to 0.73 for the SPECT readers (Table 2).

**Table 2:** Areas under the Receiver-Operator Characteristics Curves (AUC) for all readers

	**AUC - CMR**			**AUC - SPECT**			**Δ - AUC**
**Sub-study 1 (n = 238)**	**mean AUC**	**LCL**	**UCL**	**mean AUC**	**LCL**	**UCL**	**CMR vs SPECT**
Reader A	0.72	0.63	0.80	0.63	0.53	0.72	+0.09
Reader B	0.79	0.72	0.85	0.70	0.62	0.77	+0.09
Reader C	0.75	0.67	0.82	0.63	0.53	0.73	+0.12
Range for AUC	0.07	-	-	0.07	-	-	-
Sub-study 2 (n = 227)							
Reader A	0.76	0.68	0.82	0.69	0.61	0.76	+0.07
Reader B	0.83	0.77	0.88	0.69	0.60	0.76	+0.14
Reader C	0.75	0.68	0.81	0.73	0.64	0.80	+0.02
	0.08	-	-	0.04	-	-	-

Sensitivity and specificity scores for CMR (for details see reference [16]) were also calculated at the threshold of best test performance (i.e. at a summed grading of 21, see Figure 2A) and demonstrated non-inferiority for CMR for both, sensitivity and specificity primary endpoints with a lower confidence level for the difference (ΔLCL) of −0.05 and −0.08, respectively (i.e. the lower bounds of the 95% confidence intervals for the sensitivity and specificity scores fall within the equivalence margin <10% vs SPECT). At a summed grading of 19 (see Figure 1A, dot to the right on the ROC curve), CMR is read at a low threshold for perfusion deficits and achieves superior sensitivity vs SPECT (ΔLCL of +0.01), but is of inferior specificity (ΔLCL is −0.14). Conversely, at a summed grading of 23 (see Figure 2A), i.e. at a high threshold for CMR reading (grading=23), CMR is inferior for sensitivity vs SPECT (ΔLCL is −0.11), but achieves non-inferiority for specificity (ΔLCL is −0.04).

### Performance in patients with pharmacological and physical stress

During the SPECT studies, 162 patients were stressed by exercise. In this subgroup the AUC for the SPECT technique was 0.67 ± 0.04 (which was similar in comparison of the entire SPECT population with 0.65 ± 0.03), but performance was lower compared with CMR of 0.75 ± 0.04 (p = 0.035, n = 147). AUC for SPECT with pharmacological stress was 0.64 ± 0.03, which was lower compared with CMR (AUC: 0.74 ± 0.02, p = 0.0015).

## Discussion

The main results of the trial can be summarized as follows: 1). The diagnostic performance of perfusion-CMR assessed as the area under the ROC curve was superior over SPECT in detecting CAD when assessed in the entire study population, and 2). Perfusion-CMR was also superior (higher AUC) for CAD detection in the sub-groups analyzed such as in patients with gated-SPECT and non-gated-SPECT, in patients with multi-vessel disease, in men as well as in women, and in the patients without prior MI.

### Perfusion-CMR and SPECT comparison

The current MR-IMPACT II results are well in line with a previous perfusion-CMR multicenter study [3]. The mean AUC for the CM doses of 0.05 and 0.1 mmol/kg in a previous smaller multicenter study was 0.79 which is close to the 0.75 in MR-IMPACT II. Diagnostic performance in the MR-IMPACT II with an AUC of 0.75 was slightly lower than in MR-IMPACT I with an AUC of 0.86. This might be related to the larger number of participating sites in MR-IMPACT II, by which less experienced centers could have contributed to the data. Also, in MR-IMPACT II a slightly lower CM dose of 0.075 mmol/kg was used than the most effective dose in MR-IMPACT I of 0.10 mmol/kg (for regulatory purposes the lowest efficacious dose was tested). With 33 participating sites in US and Europe, this MR-IMPACT II is to our knowledge the CMR study on myocardial ischemia with the highest number of contributing centers conducted so far. Since these centers participated with various CMR systems and drop-out rate was kept as low as 5.6%, the study results are assumed to adequately reflect the performance and robustness of perfusion-CMR in the day-to-day clinical setting. Results of the so far largest single center perfusion-CMR trial called CE-MARC were published recently [9]. In CE-MARC 752 patients were recruited to undergo 3 tests, i.e. a stress perfusion-CMR, a gated-SPECT, and CXA. In the 628 patients who completed all three tests, diagnostic performance of CMR was superior to SPECT to detect >50% diameter stenosis on CXA with AUCs of 0.84 versus 0.69 (p < 0.0001). Of note, in CE-MARC performances for both, perfusion-CMR and gated-SPECT were similar to those obtained in the current multicenter MR-IMPACT II. These results of the MR-IMPACT II underline the strengths of the perfusion-CMR technique. Correction of cardiac and breathing motion with ECG-triggering and breath-holding, respectively, appears to reliably minimize motion-related artifacts as less than 6% of data had to be excluded from analyses due to inadequate quality. This approach preserves the nominally high spatial resolution of perfusion-CMR and thereby allows detecting small even subendocardial perfusion deficits. Moreover, as with any MR acquisition, the MR perfusion images are not compromised by signal attenuation artifacts which could be perceived as perfusion deficits.

SPECT imaging also proved to be robust over the 33 centers and the SPECT results of this trial are in close match which those of MR-IMPACT I with AUC of 0.65 vs 0.67, respectively. The SPECT results are also well in line with CE-MARC and other previous multicenter SPECT trials [18,21-23] with sensitivities (ranging from 77% to 87% vs CXA) and specificities (ranging from 36% to 58% vs CXA) being located very closely to the ROC curve of the current MR-IMPACT II. Of note, also higher specificities for the SPECT technique were reported in single center studies.

When comparing the AUCs for perfusion-CMR and SPECT, superiority of CMR was achieved versus the entire SPECT population (p = 0.0004, n = 425) as well as for the gated-SPECT population (p = 0.018, n = 253). When assessing the diagnostic performances (=AUCs) of both, CMR and SPECT, it should be kept in mind that the readings were performed in a fully blinded fashion without integrating any clinical information of the patients which is likely to result in underestimation of the clinical diagnostic performance of these imaging techniques.

### Performance of perfusion-CRM in multivessel disease patients, in patients without prior MI, and in men and women

As patient prognosis is coupled to the extent of ischemia, it is important to note, that the diagnostic superiority of perfusion-CMR was also preserved in the MVD patients as shown in Figure 2B.

In the study by Lin and coworkers [24], women were less likely to undergo stress testing prior to PCI compared with men and other studies demonstrated, that younger women had a worse outcome after acute MI compared to age-matched men [25]. These findings go along with particular difficulties in women for ischemia detection, as breast tissue can cause suboptimal imaging conditions. Also, women are more susceptible to radiation sequelae in terms of cancer incidence [26,27]. Therefore, a radiation-free test for ischemia detection in women is particularly valuable. The results of MR-IMPACT II demonstrate equal diagnostic performance of CMR irrespective of gender, which was superior to SPECT in both, men and women (Figure 3A/B). While multicenter data for SPECT in women are rare, one study [21] yielded 95%-confidence intervals for sensitivity and specificity of 69-100% and 10-61%, respectively, which fall onto the SPECT-ROC curve for women of the current study. Similarly, a single center study using thallium SPECT found a lower diagnostic performance in women versus men, which was related to smaller heart sizes in women [28]. As spatial resolution of perfusion-CMR is higher than in SPECT and attenuation artifacts do not occur, it is not surprising that two earlier single center studies found a similar performance of CMR in women and men [29,30], which is well in line with the current multicenter findings.

For CMR image interpretation, only the stress perfusion images were used, but the readers were exposed to the viability images obtained by applying the late enhancement CMR technique. To test, whether viability information could have played a major role in the recognition of ischemia, a sub-analysis was performed which excluded the patients with prior MI. This reduced the sample size to 174, but the difference in AUC persisted in favor of CMR (with a p-value of 0.0054).

### Limitations of the study

The definition of CAD applied in this study was primarily dependent on coronary anatomy (in most patients by fulfilling the criterion of a ≥50% diameter stenosis in a ≥2 mm vessel and in a minority of patients by the history of MI, even when the infarct-related vessel was non-stenosed after PCI). This anatomy-based definition does not consider collateral flow and microcirculatory factors that modify the hemodynamic relevance of epicardial stenoses. Nevertheless, this definition was deemed best as it is relatively easy to measure, is frequently used in such comparative studies, and it often sets the basis for patient management in clinical routine. In this context is should be noted that an optimal patient management should always consider the patient’s symptoms, his risk factor profile, and the prognosis (derived from risk factors, symptoms, and imaging information).

The aim of this study was to assess the diagnostic performance of CMR and SPECT to identify patients with CAD through the detection of perfusion abnormalities. Once CAD is identified, a further evaluation is recommended to assess scar tissue. As the late enhancement CMR technique is well documented as a robust and precise method to detect scar [31,32], this additional work-up was not tested in this trial. We would like to stress, however, that perfusion testing should be accompanied in general by a viability testing, particularly in patient with reduced LV function, to allow for an optimal patient management. To explore a potential influence of scar tissue on the study results, the ROC analysis was repeated in the patient population without prior MI and superiority of CMR over SPECT was preserved (p = 0.0054).

In this trial patients with decompensated heart failure, after bypass surgery, and with relevant arrhythmias were excluded, and thus, the findings of this study should not be applied to these patient groups. Due to the inclusion criteria, the frequency of CAD was relatively high with 48.8% in this trial. While this rather rigorous recruitment strategy represents a strength of the trial, the trial results cannot be extrapolated to other populations with lower disease prevalence, e.g. to asymptomatic screening populations.

While this study shows that perfusion-CMR is useful for the detection of CAD, further studies will be needed to address the question, whether this perfusion information when used to guide revascularizations also improves outcome in comparison to other non-invasive methods that test ischemia.

## Conclusions

This large international, multicenter, multivendor, prospective trial performed at 33 centers and including 465 patients shows perfusion-CMR as superior to gated-SPECT for the detection of CAD when comparing test performance by ROC analysis. Perfusion-CMR was also superior to SPECT in detecting CAD in men as well as in women. Perfusion-CMR can be recommended as an alternative for SPECT imaging.

## Competing interests

J. Schwitter, MD and N. Al-Saadi, MD served as consultants for GE Healthcare (former Amersham Health) and received honoraria. O. Strohm, MD, is a consultant of Circle (a software company not involved in data analysis in this trial). N. Hoebel, MSc, is an employee of GE Healthcare and was responsible for the statistical analyses.

## Authors’ contributions

JS: is responsible for the conception and design of the study, the data acquisition and interpretation, and he drafted the manuscript. CMW: contributed to the acquisition and interpretation of data and critically revised the intellectual content of the draft. NW: contributed to the collection and assembly of data and critically revised the intellectual content of the draft. NA: contributed to conception and design of the study, the acquisition and interpretation of data, and he critically revised the intellectual content of the draft. ES, KH, SOS, KD, OS, HA, TD: contributed to the collection of data and critically revised the intellectual content of the draft. NH: contributed to conception and design of the study, she performed the statistical analyses, contributed to data interpretation, and she critically revised the intellectual content of the draft. TS: contributed to conception and design of the study, the acquisition and interpretation of data, and he critically revised the intellectual content of the draft. All authors read and approved the final manuscript.

## Author’s information

**List of participating sites** (in alphabetical order)

H. Ahlstroehm, Akademiske Sjukhuset, Uppsala, Sweden; N. Al-Saadi, Universitätsklinikum Charité, Berlin, Germany; J. Barkhausen, Elisabeth Krankenhaus, Essen, Germany; D.S. Berman, Cedars-Sinai Medical Center, Los Angeles, CA, USA; J. C. Carr, NW University Medical Center, Chicago, IL, USA; T. Dill, Kerckhoff-Klinik Bad Nauheim, Germany; S. Flamm, St. Luke’s Episcopal Hospital, Houston, TX, USA; H. Frank, Donauklinikum Landeskrankenhaus Tulln, Austria; T. Fuisz, Washington Hospital Center, Washington, DC, USA; D. Hahn, Universität Wuerzburg, Germany; K. Huettl, Semmelweis University, Budapest, Hungary; C.M. Kramer, University of Virginia Health System, Charlottesville, VA, USA; H. Kuehl, Universitätsklinikum Aachen, Germany G. Layer, Klinikum der Stadt Ludwigshafen, Germany; M. Lombardi, Istituto di Fisiologia Clinica, CNR, Pisa, Italy; A. Luchner, Klinikum der Universität Regensburg, Germany; E.T. Martin, Oklahoma Heart Institute, Tulsa, OK, USA; G. P. Meyer, Medizinische Hochschule Hannover, Germany; A. Mosterd, Meander Medisch Centrum, Amersfoort, The Netherlands; E. Mousseaux, European Hospital G. Pompidou, Paris, France; S. Orn, Stavanger Hospital Trust, Stavanger, Norway; R.M. Peshock, SW Medical Center at Dallas, Dallas, TX, USA; G. Raff, William Beaumont Hospital, Royal Oak, MI, USA; N. Reichek, St. Francis Hospital, Roslyn, NY, USA; B. Rensing, SA Ziekenhuis, Nieuwegein, The Netherlands; E. Sauer, Krankenhaus Landshut, Germany; S. Schoenberg, Klinikum der Universität Muenchen-Grosshadern, Germany; J. Schwitter, University Hospital Zurich, Switzerland; U. Sechtem, Robert-Bosch Krankenhaus, Stuttgart, Germany; T. Simor, University of Science, Pecs, Hungary; O. Strohm, St. Getrauden Hospital, Berlin, Germany; A. van Rossum, VU Medical Center, Amsterdam, The Netherlands; C. Wacker, Medizinische Universitätsklinik Wuerzburg, Germany; N. Wilke, University of Florida Jacksonville, Jacksonville and Gainsville, FL, USA; P. Woodard, Mallinckrodt Institute of Radiology, St. Louis, MO, USA.

## Funding source

This work was supported by GE Healthcare (former Amersham Health).
